# Supplementary material for: Digital governance, anti-corruption and political stability: An empirical study using cross-national panel data
Source: PLoS One. 2025 Dec 17;20(12):e0338630. doi: 10.1371/journal.pone.0338630 (PMC12711073; doi:10.1371/journal.pone.0338630)
Supplement: Attachment — Submitted filename: Review.pdf [file pone.0338630.s002.pdf]

General Evaluation

1. Originality and Significance

- **Strengths**: The paper addresses a timely and relevant issue in the context of global governance, with a focus on digital governance's impact on political stability, mediated by anti-corruption efforts. It is significant because it provides empirical evidence using panel data from 112 countries between 2014 and 2023, which is a comprehensive dataset for this kind of analysis. The combination of digital governance, anti-corruption, and political stability is a well-rounded approach to understanding governance mechanisms on a global scale.

- **Suggestions**: While the study is innovative, it would benefit from a clearer distinction in how the digital governance strategies might differ in their impact across regions. The paper could explore more specific mechanisms or case studies that showcase regional variations or unique challenges to digital governance, beyond just the economic levels.

2. Methodology

- **Strengths**: The use of econometric models, such as the individual fixed effects model and the stepwise regression method for testing the mediation effects, is well-suited to address the research questions. The robustness checks and heterogeneity analysis (by income group) add strength to the empirical approach.

- **Suggestions**:
  - The methodology section could provide a bit more detail regarding the assumptions of the models. For instance, while the paper mentions that logarithmic transformations were applied to some variables to address heteroscedasticity, further clarification on how this was addressed specifically in the models and why this method was chosen would be beneficial.
  - The Sobel and Bootstrap tests for robustness are appropriate; however, it might be helpful to include a discussion of potential limitations or biases inherent in the data, such as the quality of governance data or discrepancies in the political stability index used.

3. Clarity and Structure

- **Strengths**: The paper is well-organized and the flow of information is logical. Sections like the introduction, literature review, and methodology clearly explain the theoretical foundation, research design, and data. The paper adheres to the general structure expected for academic papers in political science and economics.

- **Suggestions**: There are some minor language issues that need to be addressed for clarity. For example, terms like "political stability" could be explained further in the introduction for non-specialist readers, as well as more concrete examples of "digital governance" and "anti-corruption efforts" from real-world cases.

4. Data and Results

- **Strengths**: The results section is well-detailed, with multiple regressions and robustness tests supporting the hypotheses. The analysis of heterogeneity across

different income levels is an important contribution, highlighting the differential impacts of digital governance across countries at different stages of economic development.

- **Suggestions**:
  - The results could benefit from additional discussion on the limitations of the political stability index used. Although WGI is a widely accepted source, the potential biases in these data should be acknowledged.
  - The study could also consider including additional variables, such as regional effects or a deeper analysis of corruption types, to give a more nuanced understanding of how digital governance interacts with political stability.

5. Conclusions and Policy Implications

- **Strengths**: The conclusions are relevant, offering clear policy recommendations based on the findings. These include establishing multilateral cooperation mechanisms for digital governance, combatting transnational corruption, and creating a global policy evaluation system, all of which are practical and actionable.
- **Suggestions**: The policy recommendations could be expanded to include more detailed actionable steps for specific countries or regions. For instance, low-income countries might need more tailored recommendations on digital governance capacity-building, considering their limited technological infrastructure.

Overall Assessment

- **Strengths**: The study is a valuable contribution to understanding the role of digital governance in enhancing political stability through anti-corruption efforts. The use of cross-national panel data makes the findings robust and globally applicable. The policy recommendations are practical and timely.
- **Suggestions for Improvement**:
  - Expand on how digital governance might operate differently across various regions, particularly for countries at different income levels.
  - Provide more detailed discussion on data limitations, particularly regarding the political stability index.
  - The methodology could benefit from more transparency regarding model assumptions and choices.

The manuscript is generally well-written but could benefit from **minor revisions for clarity**, grammar, and sentence structure. It would be helpful to **simplify some of the more complex sentences** and correct any typographical errors to ensure the manuscript is fully intelligible.

Recommendation: Accept with revisions

The paper makes an important contribution to the literature and provides significant empirical insights. Major revisions, particularly in the clarity of methodology and the robustness of the data sources, would further strengthen the paper.
