# Supplementary material for: Digital governance, anti-corruption and political stability: An empirical study using cross-national panel data
Source: PLoS One. 2025 Dec 17;20(12):e0338630. doi: 10.1371/journal.pone.0338630 (PMC12711073; doi:10.1371/journal.pone.0338630)
Supplement: Attachment — Submitted filename: Response to Reviewers.docx [file pone.0338630.s003.docx]

**Dear Editor and Reviewers,**

First and foremost, i would like to extend my sincere gratitude for the time and effort you have dedicated to reviewing my manuscript. Your invaluable feedback greatly aids in enhancing the quality of my research. Now, every comment was meticulously addressed, ensuring the the necessary adjustments were made to enhance the paper’s rigor and clarity. I believe that these revisions have significantly improved the manuscript, rendering it more comprehensive and refined.

Herein, I provide detailed responses to each of the comments made by the reviewers:

**Comment 1:**

- Response: We greatly appreciate your suggestion and have made the appropriate revisions in the manuscript. We also show the revised content here.

<<<The impact of digital governance level on the degree of political stability shows regional heterogeneity. As the research sample consists of 112 countries, which vary greatly in culture, system and development stage, it is difficult to capture their complex and multi-dimensional relationships by directly conducting full sample regression or simple regional dummy variable interaction terms. Therefore, this study adopted a stratified systematic sampling method to strategically reduce the initial sample. First, we stratify the overall sample based on two dimensions highly relevant to theoretical research: 1. Geographical region. According to the United Nations geoscheme (2025), countries are divided into five continents (Asia, Europe, Africa, America, and Oceania) to control the differences in macro historical and cultural backgrounds. 2. Digital governance level. Based on the existing data, the average value (0.0004932), minimum value (-2.023), and maximum value (1.382) of digital governance levels in various countries were obtained and segmented in an equal-width manner. Countries within each continent are classified into three levels: low digital governance level (within the range of [-2.023, -0.888]), medium digital governance level (within the range of [-0.888,0.247]), and high digital governance level (within the range of [0.247,1.382]). Secondly, within each "regionally Digital governance level" cell formed by the intersection of the above two dimensions (for example, "Asia - High Digital Governance Level"), we aim to extract as many equal numbers of countries as possible. This "equal division" principle ensures that our sub-samples are not dominated by a specific group (such as European countries with high digital governance), thereby guaranteeing that each level of digital governance is evenly represented across different regions. This is crucial for fairly testing regional differences. Thirdly, through the above procedures, we ultimately obtained a highly representative balanced panel sub-sample covering all regions and digital governance levels around the world, which is approximately one-third of the original sample. We admit that this sacrifices a portion of the sample size, but we believe that this trade-off brings a key advantage to the research, namely enhanced comparability. Because if only the full sample is used, the research finds that it might be dominated by specific regions with extremely high or low levels of digital governance and large sample sizes (such as Europe or Africa), while this method effectively 'matches' countries in different regions that are at similar stages of digital governance development. For instance, we can representatively compare "high digital governance countries in Africa" with "high digital governance countries in Asia", thereby more purely identifying the "regional" effect while controlling for the variable of "digital governance level".

The regression estimation results are listed in columns (1) to (3) of Table 6. The results show that the impact of digital governance level is "context-dependent" rather than universal. In column (1) of Table 6, the impact of digital governance level is negative but not significant. This indicates that in this group of countries, digital governance levelhas not effectively promoted stability and may even have a slightly negative impact by amplifying social conflicts or being abused. Another factor that also has a negative impact but is very significant is the education level, which conforms to the assumption of the "Malthusian trap" (Grinin 2022) : In the case of an improvement in the level of education but no improvement in the opportunity structure, civic awakening may give rise to dissatisfaction with the current situation. However, the influence of the rule of law in this context is positive but not significant, indicating that in these countries, the rule of law itself may not be sound enough to serve as a stable cornerstone. Therefore, this group of countries may be confronted with deep-seated problems such as weak national capacity, incomplete systems or social divisions. The fundamental social and political issues (such as fairness, opportunity, and security) could be the primary contradictions among these countries. The "superstructure" such as digital governance is difficult to play an active role and may even exacerbate the contradictions due to improper use. For example, in this group of samples, Haiti's national conditions are consistent with this result. According to the EDGI report (2025), "As in 2022, Haiti remains at the lowest EGDI level in the region with ongoing political crises and conflicts severely undermining efforts to create a stable and effective digital infrastructure." In column (2) of Table 6, the impact of digital governance level on political stability degree is positive and significant. However, unlike the previous group of countries, the influence of the rule of law level in this group of countries is no longer significant. This does not mean that the rule of law is unimportant; rather, it indicates that the rule of law has become a common "infrastructure" in this group of countries, with a relatively small degree of variation. Therefore, the role of digital governance as an "efficiency enhancer" has been highlighted. For instance, the situation of Bangladesh in this sample is rather typical. The country is orderly moving on the path of sustainable development, integrating digital technology with people's livelihood and economic development, providing stable jobs, promoting industrial innovation and stabilizing infrastructure (ESCAP 2024). In column (3) of Table 6, the impact of digital governance level on national political stability is no longer significant. In other words, when all countries are mixed together to calculate the "average effect", the net effect of digital governance level disappears. This precisely proves that its positive effect column (2) and negative effect column (1) cancel each other out in different types of countries. This is also the most powerful evidence to prove the existence of regional differences. This result also confirms the "threshold effect". For digital governance to have a positive impact, it needs to cross a key "institutional threshold". The countries in column (1) are below this threshold. The main contradiction lies in fundamental institutional issues (such as the lack of effective rule of law and social injustice), and the effect of digital governance is not significant. The countries in column (2) have crossed this threshold and thus can reap the "digital dividend". Overall, whether the level of digital governance can enhance political stability depends on the institutional environment of the country.

**Table 6.** Heterogeneity test of digital governance levels

|  | **(1)** | **(2)** | **(3)** |
| --- | --- | --- | --- |
|  | ***Low-level digital governance*** | ***Middle-level digital governance*** | ***High-level digital governance*** |
|  | ***ln PS*** | ***lnPS*** | ***lnPS*** |
| *DG* | -0.202 | 0.243*** | 0.028 |
|  | (-0.4064) | (2.5775) | (0.1902) |
| *el* | -0.024*** | 0.009 | -0.001 |
|  | (-4.6988) | (1.0501) | (-0.3346) |
| *ln ps* | 0.010 | -1.853** | -1.076* |
|  | (0.0073) | (-2.5318) | (-1.8266) |
| *ln rl* | 0.176 | 0.041 | 0.218 |
|  | (0.8602) | (0.2256) | (0.7199) |
| *cons* | 2.220 | 36.571*** | 21.710** |
|  | (0.0912) | (2.7312) | (2.0444) |
| Individual fixed effects | Yes | Yes | Yes |
| Time fixed effects | No | No | No |
| Sample size | 97 | 121 | 152 |
| R-squared | 0.916 | 0.928 | 0.952 |

**Comment 2:**

- Response: Your point is indeed pivotal, and we have adjusted the manuscript in line with your recommendation. We also show the revised content here.

Comment 2.1

<<<Although the individual fixed effects model effectively addresses a major source of omitted variable bias by eliminating individual characteristics that do not change over time, such as a country's historical culture and geographical environment, it cannot automatically guarantee that the error terms are homoscedasticity. The heteroscedasticity in the sample data of this study may be manifested as different variances of error terms in different countries (individuals). If heteroscedasticity is ignored, although the coefficient estimator remains unbiased and consistent, its standard error will be biased, which may lead to inaccurate confidence intervals. Therefore, we adopted a multi-level processing strategy: 1. Perform logarithmic transformation on the variables to alleviate skewed distribution and extreme values; 2. Using Cluster-Robust Standard Errors to deal with any form of intra-group correlation, that is, allowing for any form of correlation and heteroscedasticity among error terms within individuals (different years in the same country). >>>

Comment 2.2

<<<Although this study employed robust statistical tests such as Sobel and Bootstrap to identify mediating effects, a thorough discussion of the inherent limitations of the data itself is crucial for the correct interpretation of the research conclusions.>>> A more in-depth discussion is shown in the reply to Comment 4.1.

**Comment 3:**

- Response: Your are correct in this regard. Upon re-evaluation, we have corrected the said oversight. We also show the revised content here.

Definition of political stability<<<According to Hurwitz (1973), political stability can be defined based on five aspects: (1) Stability refers to the absence of violence; (2) Stability refers to the durability of a government's lifespan; (3) The stability of its existence as a legitimate constitutional order; (4) Stability of the structure without change; (5) Stability as a multi-faceted social attribute.>>>

Examples of digital governance<<<Digital governance aims to utilize digital technologies and data resources to govern and manage various fields such as the economy, society, and government. Its application scope is constantly expanding, and an increasing number of countries are incorporating it into their governance frameworks. For instance, Uruguay has launched the firma.gub.uy website, offering advanced electronic signature services from multiple providers registered with the electronic certification department. This is convenient for individuals and enterprises to use and verify, aiming to support all online cross-border transactions, save users' time and money, simplify administrative processes, reduce transaction obstacles, and enhance the productivity and competitiveness of enterprises; also, Singapore's application of artificial intelligence in public services has enhanced service efficiency in the healthcare and transportation sectors (EGDI 2025). >>>

Cases of anticorruption efforts<<<For example, Russia has established competitive procurement procedures in electronic form on electronic platforms, allowing the use of artificial intelligence technology to detect economic anomalies and signs of formal competition in government contracts. Meanwhile, since January 1, 2021, cryptocurrencies have been regarded by many countries as objects of anti-corruption monitoring (Sergey 2020).>>>

**Comment 4:**

- Response: We’ve considered your perspective and partially adopted your recommendation, implementing pertinent modifications in the paper. We also show the revised content here.

Comment 4.1

<<<Although this study employed robust statistical tests such as Sobel and Bootstrap to identify mediating effects, a thorough discussion of the inherent limitations of the data itself is crucial for the correct interpretation of the research conclusions. First, the measurement of digital governance level faces inherent challenges. Many mainstream digital governance indices (such as the UN's EGDI) rely on expert surveys and business questionnaires. These data often reflect the "perception" of business executives or specific expert groups, rather than the actual usage experience of ordinary citizens or the objective effectiveness of the government's back-end operations. This "elite perspective" may lead to a systematic overestimation of the governance levels of countries that have invested more in digital propaganda but have insufficient actual inclusiveness. Meanwhile, existing indices usually focus on the breadth of online service supply and digital infrastructure, but they do not adequately measure the "quality" dimension of digital governance. As a result, our research may capture the "efficiency" aspect of digital governance, but fail to fully capture its "fairness" or "rights" aspect, the latter of which may have a more complex relationship with political stability. Second, the degree of political stability encompasses various aspects. For instance, the "political stability and no violence/terrorism" indicator in the WGI used in this study. However, such measurements may focus more on "the stability of order" or "the stability of regime", and the capture of "the stability of the system" - that is, the long-term resilience and legitimacy of the political system itself - is relatively indirect. Therefore, our research finds that digital governance can promote stability, but the nature of this stability requires careful interpretation. The limitations of the above-mentioned data are not the fatal weakness of our research, but rather a common challenge faced by all cross-border quantitative political economy studies. They mainly affect the accuracy of our conclusion and the boundary conditions. Therefore, our conclusion should be understood as -- there is a connection between digital governance and political stability through a specific mechanism under the current mainstream measurement system.>>>

Comment 4.2

We appreciate the reviewer's valuable suggestion to delve deeper into variables like regional context or corruption typologies. We agree that these factors are intriguing and merit scholarly attention.

The primary focus of our current manuscript, however, is to establish the foundational, cross-national relationship between digital governance and political stability at a macro level. Introducing highly context-specific variables, while valuable, would shift the scope of our paper towards a comparative case study analysis, which is beyond its central aim. We were concerned that such an expansion might dilute the clarity and parsimony of our core theoretical model and empirical tests.

Instead, we have strengthened the Discussion section by incorporating the reviewer's idea as a key direction for future research. We now suggest that subsequent studies could build on our findings by conducting focused comparisons within specific regions or by employing qualitative methods to unpack the nuances of different corruption types.

**Comment 5:**

- Response: We thank you profoundly for your detailed insights, which have been instrumental in refining our work. We also show the revised content here.
- <<<Fourth, Countries at different digital governance level should implement different countermeasures. Firstly, for the groups in this study where the digital governance effect is not significant or even negative, the core challenges for these countries are the digital divide and the insufficiency of national basic capabilities. Therefore, these countries can prioritize investment in mobile network coverage, skip the large-scale deployment of expensive fixed broadband, focus on high-impact and low-complexity pilot projects, achieve rapid results, and accumulate public trust. Also, they could concentrate the limited investment in digital governance on the known areas with a high incidence of corruption, allowing citizens to see the improvements brought about by digital governance. Secondly, for the groups with significant digital governance effects in the results of this study, the goals of these countries should be "connection" and "empowerment". Specifically, these countries can break down the "data silos" among various departments through legislation or by establishing systems, and prioritize the formulation of data sharing standards. At the same time, they could establish a supervised testing environment for digital governance innovation. This can not only encourage innovation but also prevent new social conflicts caused by immature technology or design flaws, balancing efficiency and stability. Thirdly, for the countries with high levels of digital governance but insignificant influence in the results of this study, the challenge lies in the upgrading of technological ethics and governance paradigms. These countries should establish regulations requiring that the automated decision-making systems used by their governments must meet the requirements of transparency, interpretability and human supervision, and prevent "black box" algorithms. In conclusion, considering the differences in economic and digital governance levels among countries, an effective digital governance strategy must be diagnostic. For low-income countries, it is a catalyst for development; For middle-income countries, it is a booster for institutional reform; For high-income countries, it is the guardian of social resilience. Decision-makers should first assess the stage and core challenges their country is at, and then select the policy toolkit that matches them for customized implementation.>>>

All revisions have been made with respect to the feedback received, and we hope they meet your expectations. Once again, thank you for your constructive critique.

In conclusion, we hope the revised manuscript will be deemed suitable for publication. We eagerly await your further comments or decision.

Sincerely,

Yaxing Zhao, Zhizhou Du
