# Supplementary material for: Digital governance, anti-corruption and political stability: An empirical study using cross-national panel data
Source: PLoS One. 2025 Dec 17;20(12):e0338630. doi: 10.1371/journal.pone.0338630 (PMC12711073; doi:10.1371/journal.pone.0338630)
Supplement: Attachment — Submitted filename: Reviewer_Letter_2.docx [file pone.0338630.s004.docx]

**Reviewer Letter Manuscript Title: Digital governance, anti-corruption and political stability: an empirical study using cross-national panel data**

Thank you for the opportunity to review the revised version of this manuscript. I have carefully examined the authors’ response letter and the revised manuscript. Below, I provide an expanded, analytic evaluation assessing whether the authors have sufficiently addressed the concerns raised in the prior review round.

**1. Originality, Significance, and Theoretical Framing**

The revised manuscript demonstrates meaningful progress in addressing concerns regarding regional differentiation and the contextual mechanisms through which digital governance influences political stability. The addition of a two-dimensional stratification approach—based on UN regional groupings and digital governance levels—introduces a more nuanced analytical frame. The authors also provide a clearer theoretical justification for why digital governance may yield heterogeneous political outcomes depending on institutional maturity, bureaucratic capacity, and regional norms. These revisions significantly enhance the conceptual robustness of the study.

**2. Methodology, Model Assumptions, and Analytical Transparency**

The authors have strengthened the methodological rigor by clarifying model assumptions, addressing heteroscedasticity, and explaining the use of logarithmic transformations. The explicit justification for cluster-robust standard errors and the improved transparency in the description of the mediation analysis (including the Sobel and Bootstrap procedures) address previous concerns effectively. The analytical sequence is now well-articulated, offering a replicable and logically coherent empirical strategy.

**3. Clarity of Key Concepts and Use of Illustrative Examples**

The revised manuscript incorporates precise definitions of political stability, digital governance, and anti-corruption grounded in authoritative literature. The incorporation of concrete country examples—such as Uruguay, Singapore, and Russia—provides real-world grounding that enhances clarity and broadens the manuscript’s accessibility to readers not specialized in governance studies. This improves the pedagogical quality of the manuscript and strengthens its fit for a multidisciplinary journal such as PLOS ONE.

**4. Data Quality, Measurement Limitations, and Scope of Variables**

A major improvement in the revised version is the explicit acknowledgment of measurement limitations associated with the WGI political stability index and other governance indicators. The authors discuss potential biases, elite-driven valuation effects, and conceptual risks inherent in governance metrics. Their justification for not expanding the model to include corruption typologies or region-specific fixed effects is well-reasoned, based on the macro-comparative objective of the study. This addition demonstrates an appropriate degree of methodological humility and transparency.

**5. Interpretation of Results and Depth of Policy Implications**

The revised manuscript now offers a more detailed, stratified interpretation of empirical results across different income groups and digital governance environments. The policy section is substantially strengthened, providing differentiated recommendations tailored to the institutional and technological maturity of various country categories. These refinements improve the paper’s practical relevance and align the policy analysis more closely with the empirical findings.

**6. Language Quality, Structure, and Overall Presentation**

The authors have made noticeable improvements in language clarity, structural coherence, and conceptual flow. While minor editorial refinements may still be beneficial at the copyediting stage, the manuscript now reads clearly and professionally.

**Overall Recommendation**

The authors have fully addressed the major concerns raised in the previous review. The manuscript is now methodologically robust, conceptually clearer, and substantively more informative. I recommend acceptance with only minor editorial polishing.
